# Building a Personalized Medicine Infrastructure for Gynecological Oncology Patients in a High-Volume Hospital

**DOI:** 10.3390/jpm12010003

**Published:** 2021-12-21

**Authors:** Nicolò Bizzarri, Camilla Nero, Francesca Sillano, Francesca Ciccarone, Marika D’Oria, Alfredo Cesario, Simona Maria Fragomeni, Antonia Carla Testa, Francesco Fanfani, Gabriella Ferrandina, Domenica Lorusso, Anna Fagotti, Giovanni Scambia

**Affiliations:** 1 Department of Woman and Child Health and Public Health, Fondazione Policlinico Universitario A. Gemelli IRCCS, 00168 Rome, Italy; nicolo.bizzarri@policlinicogemelli.it (N.B.); francesca.sillano@policlinicogemelli.it (F.S.); francesca.ciccarone@policlinicogemelli.it (F.C.); simona.fragomeni@policlinicogemelli.it (S.M.F.); antoniacarla.testa@policlinicogemelli.it (A.C.T.); francesco.fanfani@policlinicogemelli.it (F.F.); mariagabriella.ferrandina@policlinicogemelli.it (G.F.); domenica.lorusso@policlinicogemelli.it (D.L.); anna.fagotti@policlinicogemelli.it (A.F.); giovanni.scambia@policlinicogemelli.it (G.S.); 2 Department of Life Sciences and Public Health, Università Cattolica del Sacro Cuore, 00168 Rome, Italy; 3 Scientific Directorate, Fondazione Policlinico Universitario A. Gemelli IRCCS, 00168 Rome, Italy; marika.doria@policlinicogemelli.it (M.D.); alfredo.cesario@policlinicogemelli.it (A.C.)

**Keywords:** gynecologic oncology, patient-centered care, personalized medicine

## Abstract

Gynecological cancers require complex intervention since patients have specific needs to be addressed. Centralization to high-volume centers improves the oncological outcomes of patients with gynecological cancers. Research in gynecological oncology is increasing thanks to modern technologies, from the comprehensive molecular characterization of tumors and individual pathophenotypes. Ongoing studies are focusing on personalizing therapies by integrating information across genomics, proteomics, and metabolomics with the genetic makeup and immune system of the patient. Hence, several challenges must be faced to provide holistic benefit to the patient. Personalized approaches should also recognize the unmet needs of each patient to successfully deliver the promise of personalized care, in a multidisciplinary effort. This may provide the greatest opportunity to improve patients’ outcomes. Starting from a narrative review on gynecological oncology patients’ needs, this article focuses on the experience of building a research and care infrastructure for personalized patient management.

## 1. Introduction

The concept of personalized medicine (PM) (as intended in the broader framework of “P4 Medicine” [predictive, participative, preventive, and personalized]) assumes a systemic approach to the patient, embracing multi-omic strategies, new biotechnologies, and artificial intelligence solutions, to provide “the right treatment, to the right person, at the right time” [1,2,3,4]. Indeed, although some diseases are closely linked to genetic and genomic alterations, each patient is unique; our biological predisposition to a disease is intertwined with complex and layered molecular, cellular, and systemic networks as the body adapts through several exposomes (e.g., nutrition, environment, lifestyle, emotions, etc.) in a lifetime [1].

Gynecological cancers require complex management because patients have specific needs to address. As oncological patients experience physical and nonphysical symptoms [5], it is crucial to understand their explicit and implicit needs, to provide the best personalized experience while achieving standards of care. It has been demonstrated that centralization to high-volume centers improves the oncological outcomes of patients with gynecological cancers [6,7]. Moreover, the centralization of cancer management and treatment is essential to guarantee a high-quality standard of care, as well as to allow clinical and laboratory research development and ensure a high level of clinical and surgical training to residents and fellows [8,9,10,11].

While research on PM in gynecologic oncology mostly focuses on the importance of multi-omic explorations of disease pathways, the goal of this article is to elucidate the major needs of gynecological cancer patients-who may encounter several struggles during their journey—while illustrating the case of a research hospital for centralized care, as an endeavor to achieve the holistic, integrated personalization of care in this field.

## 2. Tailoring and Centralizing Care According to Patients’ Needs

**Figure 1:**
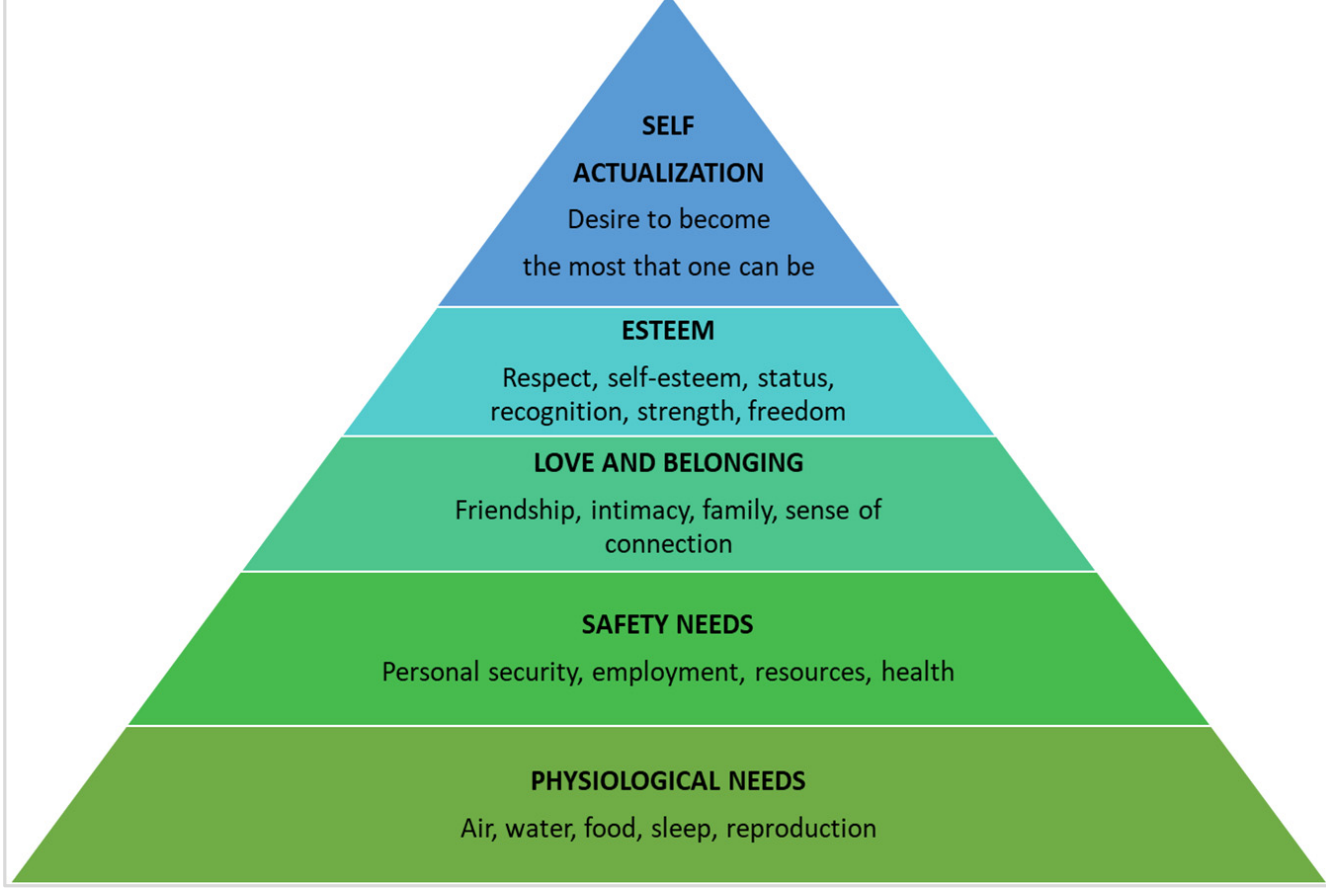
Abraham Maslow’s pyramid on the five human needs, with the most basic at the bottom.

Considering Maslow’s theory on basic human needs [12] (Figure 1) and according to the literature, gynecological cancers can affect several aspects of a patient’s life, such as [13,14,15]:

– Quality of sleep, variation in their nutritional plan, physical symptoms (e.g., feeling pain, fatigue, nausea, constipation), and changes related to the reproductive system (physiological needs);
– Uncertainty about the future, the possibility of continuing to work or resigning because of health issues that affect personal safety, the availability of financial resources to meet treatment costs, and possibly travel and lodging expenses (safety needs);
– Family and social distress with respect to roles, functions, and relationships of proximity and intimacy. These aspects redefine the sense of closeness with others (love and belonging needs);
– Self-esteem, emotional distress (e.g., anxiety, fear of recurrence), cognitive changes, need for control, and changes in social/professional status (esteem needs);
– Meaning of life, personal purpose, redefinition of self and priorities (hopes, desires and ambitions, inner strength), and meaning of illness (self-actualization needs).

These needs are explored in more detail in the following paragraphs.

### 2.1. Physiological and Safety Needs

The first set of human needs affected by gynecological malignances is physiological needs. According to the European Society for Medical Oncology [16], knowledge of cancer and tissue characteristics allows clinicians to identify the best treatment for the patient. The challenge of this era is to pursue the identification and application of innovative targeted therapies, towards cutting-edge research on precision medicine and clinical trials of novel and experimental treatment options, specifically selected according to the patient’s cancer biology or drug resistance mechanisms. On one hand, patients may face the pre-existence of several pathological situations, such as premalignant conditions (e.g., endometrial hyperplasia, cervical dysplasia) or comorbidities (e.g., obesity, ageing) [17]. On the other hand, patients have to cope with possible physical morbidities (e.g., urinary/intestinal problems) [13], and other treatment-related consequences (sexual dysfunction, infertility, lymphedema, chemotherapy-induced nausea, hair loss, fatigue) [18,19] or experimental therapies (e.g., drug toxicity) that may occur. Innovative medical approaches developed over the past decade have improved oncologic outcomes by reducing treatment side effects [18]; in fact, the risks of drug resistance and adverse events are minimized based on the specific patient phenotype.

Physiological needs also affect patients’ families in terms of cancer inheritance, especially those with a known germline pathogenic (or likely pathogenic) variant associated with increased cancer risk [20]. Patients diagnosed with cancer during pregnancy have a physiological need for safe childbirth while preserving their health and that of their child [21]. Similarily, gynecologic oncology patients may feel a desire for motherhood after cancer treatment [22]. According to the American Society of Clinical Oncology (ASCO) [23], clinicians have to identify the best treatment option for fertility preservation. In the challenge of preserving healthy tissues next to the tumor, radiation oncologists can irradiate target cells while preserving surrounding areas to avoid long-term toxicity [24]. Robotic-assisted surgery is likely to help surgeons manage patients with high BMI with a minimally invasive approach (meaning shorter hospitalization and less pain) [25]. Personalized treatments can also be integrated with alternative approaches (e.g., palliative and supportive care) to provide the relief of cancer-related symptoms in the end-of-life setting. To this aim, patients may positively benefit from meditative sessions [26,27], music therapy [28], and acupuncture [29].

Safety needs include the availability of financial, organizational, and informational resources to manage their disease. As mentioned, centralization of oncology management and treatment is essential to pursue a high quality of care [8,9,10,11]. Patients may experience disruptions in continuity of care and difficult transitions between diagnosis, treatment, and follow-up; therefore, patient-centered interprofessional practices should optimize cancer treatment [30].

Connecting the referral center to the community can lead to improved networks that support the continuity of patient care. Local and out-of-region patients have different safety needs, as travelling for a long distance to stay in the hospital has an economic impact that may influence patients’ choice of their referral center [31]. Moreover, the recent COVID-19 pandemic has called for a redefinition of care planning [31,32], especially to reduce hospitalization that could increase the in-hospital spread of the virus [33]. It is therefore important for patients to seek practical community information to plan the management of their care, such as:

– The organization and coordination of patient care and information on how and to whom to report symptoms [34];
– Gaining awareness of what community resources and services are available to help patients managing their disease [13,35,36];
– The need for information about risk of recurrence, prognosis, and late and long-term effects of treatment [13,34,37].

In line with this, healthy women also participate in screening programs (e.g., Human Papillomavirus (HPV), mammography etc.) [38], and high-risk patients may undergo genetic testing (e.g., BRCA1-2, Lynch syndrome, etc.).

### 2.2. Love and Belonging, Esteem, and Self-Actualization Needs

Oncologic diseases, including gynecologic tumors, can affect a woman’s psychological, social, and professional journey [39]. Cancer unconsciously challenges well-established cultural values (e.g., beauty, femininity, sexuality, women’s role/value, self-esteem, etc.) [40]. Patients experience psychological reactions to gynecological cancer symptoms [41]. For example, patients after gynecological cancer treatment may experience abrupt shifts in self-identity [42,43] due to the loss of physical integrity, changes in anatomy, and distancing in intimate relationships [44]. Even when expected, chemotherapy-induced alopecia affects women’s perception of femininity [45,46,47], such as feelings of shame and social stigma [48]. When patients experience psychological distress during or after cancer treatment, they require prompt availability of psycho-oncological care [49]. Healthcare professionals, such as nurses and care managers, could routinely monitor for distress and other concerns, while social workers, mental health professionals, and chaplains could help patients to understand their illness experience and provide comfort to the patient as they cope with uncertainty and burden [13]. Psychotherapy, meditation/relaxation practices, and community support could be effective in reducing patients’ anxiety and depression [13,50,51].

In a study on patients’ perception of cancer treatment, Carelle and colleagues cited family and partner affection as a side effect, especially when therapeutic choices involve fertility preservation [5]. This issue is also relevant when asking patients to give their consent to participate in clinical trials [52], particularly in an era when patient empowerment is increasingly used in cancer care [53]. Consequently, families can be guided in understanding family cancer risk to improve screening rates, self-care, and prevention activities [54]. Clinicians should also discuss with patients the meaning of genetic test results and the impact on their current and future health [55]. Genetic counselling about *BRCA1* and *BRCA2* gene mutation could help prevent future cancer for patients or their family members [56], with potential consequences for marriage and procreation [57,58].

In connection with physiological and safety needs, local educational programs should focus on educating people about the risk of gynecological cancer, prevention, and early detection to ensure effective treatment, as well as to improve body image [59].

## 3. Infrastructure of the Gynecologic Oncology Unit at Fondazione Policlinico Universitario Agostino IRCCS

Fondazione Policlinico Universitario Agostino Gemelli (FPG) is one of the largest academic hospitals in Italy with a strong oncological vocation [60]. In 2018, FPG was recognized as a “research hospital” (Istituto di Ricovero e Cura a Carattere Scientifico (IRCCS)) by the Italian Ministry of Health, for the disciplines of Personalized Medicine and Innovative Biomedical Technologies, and in July 2021, the hospital obtained Joint Commission International (JCI) accreditation for clinical best practice. Being a research hospital means that preclinical, translational and clinical research are intertwined within clinical practice.

The management of complex clinical scenarios requires a multidisciplinary effort [17] and centralization of care in order to provide holistic care. The Gynecologic Oncology unit at FPG is one of the major referral centers for gynecological cancer patients in Italy and Europe, where the integration of services and competencies is crucial to meet patients’ needs. The following paragraphs show our clinical experience on the organization of the patient’s journey according to the generic and specific needs of the patient; possibilities for personalized medicine research in this field are also illustrated.

### 3.1. Referral

**Figure 2:**
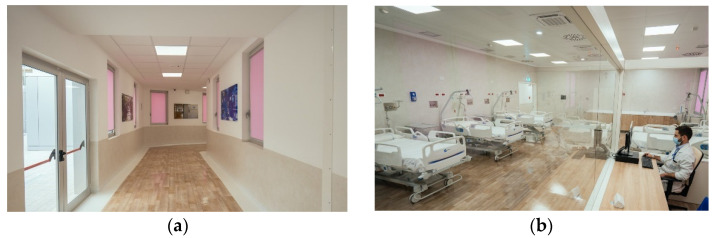
Hall (**a**) and interior (**b**) view of the emergency admission service for patients with suspected or diagnosed gynecological cancer.

Patients are usually referred by their physician to a referral center for prevention, diagnosis, treatment, and/or enrollment in clinical trials. Dedicated gynecologic oncologists examine patients with suspected or diagnosed gynecological cancers who are referred to FPG with a median time from referral to clinic of <7 days. In case of emergency, patients with suspected or diagnosed gynecological cancers have access to a specific 24/7 emergency inpatient service (Figure 2) with a gynecologic oncologist on day and night shift; a short-term observation ward is also part of this unit. The Gynecological Emergency Room was born from the need to create a dedicated pathway (“Pink Code. Women’s Oncological Path”) for women with gynecological cancer who find themselves in an emergency situation. Dedicated rooms allow doctors to offer prompt and qualified assistance, while reducing the waiting time in the General Emergency Room.

After the initial triage, performed in the General Emergency Room, women with gynecological diseases, oncological or not, are referred to the “pink area” where they are evaluated by the gynecological oncologist on duty in the emergency room who, if necessary, can discharge them, hospitalize them, or observe them in Temporary Observation (24 h) or in Short Intensive Observation (36 h), equipped with six beds.

### 3.2. Tumor Board

Tumor boards are multidisciplinary meetings dedicated to the management of specific oncological diseases. All tumor boards belong to the FPG Comprehensive Cancer Center which has recently been included in the network of the Organisation of European Cancer Institutes (OECI) with the aim of starting the accreditation procedure. All patients undergo a multidisciplinary discussion. Cancer patients have access to an initial medical examination at FPG through a dedicated service called “Front Office Assistance for Cancer Patients”.

Gynecologic Oncology boards (ovarian and uterine cancers) are made up of several specialists (e.g., gynecologic oncologist, pathologist, radiologist, radiotherapist, care manager and psychologist) who meet weekly to discuss clinical cases and make shared decisions on the most appropriate diagnostic and therapeutic options for each patient. Committed pathologists and radiologists are crucial in the diagnostic process. A specific psycho-oncologist analyzes each case and identifies the patients who need more support. A dedicated care manager takes care of the patients from the moment they are referred to our gynecologic outpatient clinic to the post-treatment period.

The patient is accompanied throughout the therapeutic process, from diagnosis to preoperative evaluation, hospitalization, and post discharge. The patient is provided with contacts to communicate with dedicated medical staff as needed during each phase. Dedicated care managers for each area (ovarian, endometrial, cervical and vulvar cancer) ensure each patient receives adequate information in the preoperative period, during hospitalization, and after hospital discharge; patients are provided with the care manager’s contact information in case of concerns at any point in their clinical management. The anesthesiology and intensive care team guarantees that radical surgery can be performed even in patients with poor performance status [61].

### 3.3. Integration of Services for Gynecological Cancer Patients

**Figure 3:**
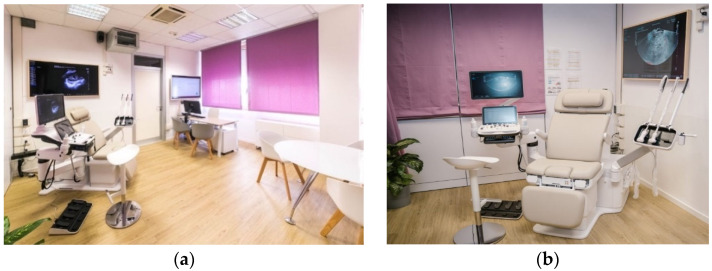
(**a**,**b**) Interior view of CLASS Ultrasound.

Candidates for surgical treatments undergo a preoperative assessment which includes blood tests, anesthetic, and gynecologic examination. Chest radiography is scheduled for those patients who do not have a CT scan of the chest, while an abdominal and transvaginal ultrasound scan is always performed since it offers a noninvasive evaluation of the risk of malignancy of ovarian masses [62], local infiltration of the cervix and endometrial cancer [63,64], and the morphology of inguinal lymph nodes in vaginal and vulvar cancer [65]. A specific department, Catholic Laparoscopy Advanced Surgery School (CLASS) Ultrasound (Figure 3), is dedicated to this task. It was built in December 2014, with three latest generation ultrasound systems (including beamforming) and three rooms with ultra-specialized staff. The goal is to perform an accurate preoperative assessment to ensure adequate neoplasm identification, histological characterization, and assessment of disease extent. A team dedicated to histological characterization by ultrasound-guided biopsy is also part of this facility. Furthermore, intra-operative ultrasound support is used on a daily basis if the surgeon needs to identify small lesions or confirm the removal of all preoperatively diagnosed disease [66].

All patients are managed under the Enhanced Recovery After Surgery (ERAS) program to decrease the length of hospitalization, perioperative complications, and costs without increasing readmission or mortality rates after surgery [67]. Patients with gynecologic malignancies treated with radiation therapy can benefit from innovative technologies and minimally invasive techniques to minimize pain and scarring on the body for patient self-perception, such as [68,69]: Magnetic-Resonance-Guided Radiation Therapy (MRI-guided) radiotherapy for treatment of moving targets; Intensity-Modulated Radiation Therapy (IMRT) and Volumetric Modulated Arc Therapy (VMAT); Stereotactic Radiotherapy (SRT); and Whole-Body Radiotherapy (WBR). Imaging is fully integrated in the initial work up, as a completely integrated preoperative work up (dedicated ultrasound and/or CT scan and/or PET/CT scan, and/or MRI DWI, etc.) which allows the estimation of the clinical stage of the disease and enables the identification of patients suitable for minimally invasive surgery or, in the case of advanced disease, for radio/chemotherapy [62].

**Figure 4:**
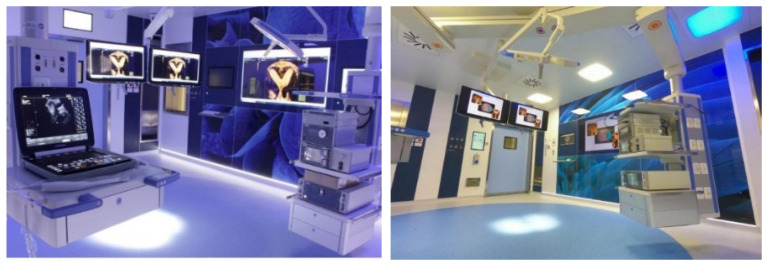
A glimpse into the rooms of CLASS Hysteroscopy.

In December 2020, the Gynecologic Oncology unit implemented an integrated digital hysteroscopy center (CLASS Hysteroscopy) with three integrated rooms equipped with advanced technologies for endoscopic imaging and tridimensional echography, as well as several technologies for intrauterine pathologies treatment (Figure 4).

The goal of this center is to move beyond the classical model (in which patients must travel multiple times to the hospital for ultrasound, diagnostic hysteroscopy, preoperative assessment, and then operative hysteroscopy in the surgical theater), towards an integrated model of digital hysteroscopy specifically designed to ensure maximum effectiveness for both patients and health professionals, particularly in the COVID-19 era, when home-to-hospital transfer may be limited. Intrauterine diseases can be treated at the same time, thanks to three-dimensional ultrasound scans, miniature instruments and endoscopes that guarantee precise intrauterine interventions guided by ultrasound images.

Concerning patients’ body image, collaboration with the plastic surgeon is established according to the size of the expected defect after radical surgery for patients with vulvar cancer. The use of flaps can reduce the risk of wound dehiscence [70].

**Figure 5:**
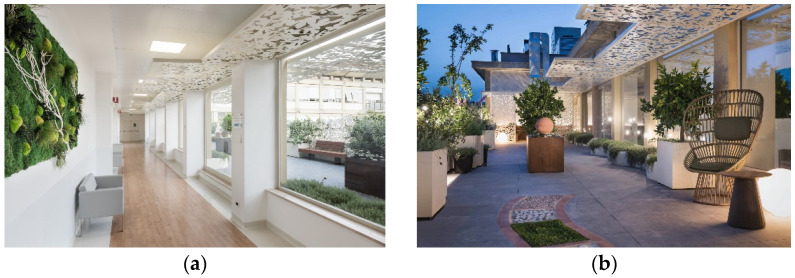
Interior (**a**) and exterior (**b**) view of the therapeutic garden.

Regarding clinical trials, patients can access phase I to phase III trials usually based on innovative target therapies [71]. A molecular care manager is responsible for tracking the results of molecular tests performed on tumor tissue and/or on blood samples. A dedicated care manager arranges patients’ appointments in the chemotherapy day unit by playing a pivotal role between patients and clinicians to manage chemotherapy side effects. Patients have access to integrated treatments and body care during treatment. Music therapy, acupuncture, and meditation sessions are provided, while body massages can be received in a specifically designed area with a therapeutic garden (Figure 5).

A dedicated team of pain therapists cares for patients as needed both at the time of diagnosis and in case of disease recurrence. As per ERAS procedures, pain control improves treatment compliance and outcome [67]. Eventually, the patient begins a palliative care path with a dedicated team, either at home or in a referral structure. Each patient can also benefit from spiritual guidance and psycho-oncological support at various points in her clinical management, from preoperative to postoperative, thanks to dedicated psycho-oncologists who provide individual psychotherapy for preassessment, outpatient, the gynecologic oncology ward, and the day hospital. Family counselling is available upon request. The presence of chaplains is an option for spiritual guidance and care. When necessary, a nutritionist is involved to create a personalized nutritional plan. Moreover, cultural mediation can be provided when necessary (e.g., to help patients understand clinical trial protocols).

Adequate postoperative management is crucial to detect potential complications early and allows rapid access to adjuvant therapies. After discharge, weekly appointments with specialized personnel are scheduled as needed until surgical wounds are completely healed. Postoperative care managers assess the postoperative course of all gynecologic oncology patients who undergo surgery at 30 days post operation; in the case of any postoperative complication, a clinical examination is scheduled with the consulting gynecologic oncologist who operated on the patient. Patients are given the direct number of the postoperative care manager should they face any symptoms in the early postoperative period.

**Figure 6:**
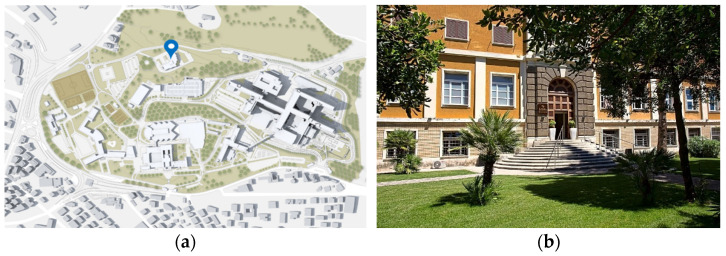
Map of the hospital campus (**a**) and external view of the hotel (**b**). The blue pin in figure (**a**) indicates the hotel location within the hospital campus (whose area is represented in color).

Patients who are referred from outside the region may benefit from a hotel stay when they need in-depth diagnostics in the prehospital phase or during the first days after hospital discharge, particularly in cases requiring short-term clinical reassessment. A perioperative care professional manages the patient’s journey to guarantee coordination and continuity of care, including communication between the referring physician on the Gynecologic Oncology unit and the hotel manager to schedule patients’ access in the prehospitalization or postdischarge phases. The hotel is located within the hospital campus (Figure 6).

Follow-up after the end of treatment is individualized according to patients’ risk of relapse. Patients at higher risk of relapse are encouraged to attend with higher frequency.

### 3.4. Personalized Medicine for Specific Needs

For a more personalized approach, the tumor board adopts clinical solutions for specific needs, as shown in Table 1.

The recent COVID-19 pandemic represented a worldwide challenge in patient management, even for high-volume hospitals. Despite this, it was fundamental for our unit to meet patients’ needs by supporting their journey through centralized care (Table 2).

### 3.5. Possibilities for Personalized Medicine Research

The strong vocation of FPG for personalized and precision medicine enables a continuous integration of molecular and clinical research with patient data and clinical outcomes. With this aim, the centralization of research facilities is also essential for our hospital.

The entire process of ensuring compliance with the approved trial protocol, ethics approval conditions, and study monitoring is supervised by a dedicated team of investigators and research support staff. The clinical research coordinator (CRC) coordinates the various phases of clinical trials. The data manager manages clinical data from the collection phase to the processing and treatment phase. The data entry manager enters clinical data into clinical research forms (CRFs) and databases. The research nurse coordinates nursing care by integrating experimental research activities with clinical practice. Specifically, a research nurse dedicated to gynecologic oncology assists clinicians on a daily basis in the decision-making phase of patient enrollment and follows patients through the therapeutic process and after discharge. Other members involved in the research are a research project manager, data scientists, a language mediator, research technicians, and a research pharmacist.

**Figure 7:**
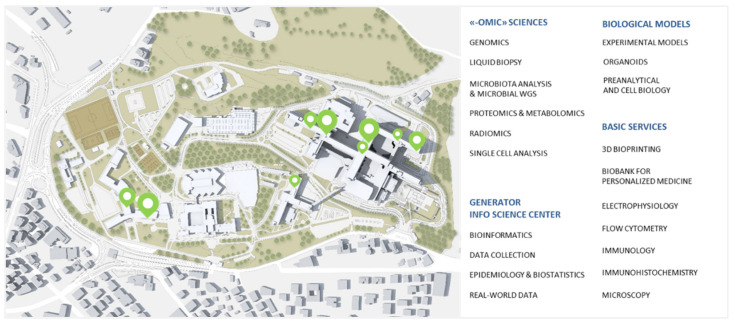
Research facilities of the Gemelli Science and Technology Park. A short virtual tour of the G-STeP can be taken at: https://gstep.policlinicogemelli.it/#/ChiSiamo (accessed on 9 October 2021).

Researchers have access to sophisticated technologies and highly specialized resources to maximize their research projects. A science and technology park (Gemelli Science and Technology Park (G-STeP)) with more than 20 research facilities has been developed since 2020 (Figure 7).

A custom, safe-by-design web application was also created by the FPG information and communications technology (ICT) division to ensure the accessibility and traceability of the entire process (https://gstep.policlinicogemelli.it accessed on 9 October 2021). It is a user-friendly platform that is able to create connections among different researchers and increases communication opportunities between specialists from different clinical and preclinical areas. The app helps researchers access different scientific services provided by each facility and provides a unique way to manage all research services. Offering an in-house research platform has several advantages, such as easy access to technology and equipment, facilitating data analysis and reducing study time.

## 4. Conclusions

The goal of the Gynecologic Oncology department at FPG is to make every therapeutic moment a research opportunity and every research effort an attempt to improve standards of care. Treatment of gynecological cancers is improving with modern technologies for the comprehensive molecular characterization of tumors and individual pathophenotypes. Ongoing studies are focusing on personalizing gynecological cancer therapies by integrating information through genomics, proteomics, and metabolomics with the patient’s genetic makeup and immune system [80,81]. Pharmacogenetic tests and therapeutic drug monitoring may be potentially beneficial for improving patient’s QoL [82], as well as the presence of a clinical pharmacologist in the multidisciplinary team for cancer care [83]. Hence, there are several challenges that need to be addressed to provide holistic benefit to the patient. Personalized approaches should also recognize the unmet needs of each patient to successfully deliver on the promise of personalized care in a multidisciplinary effort. This may provide the greatest opportunity to improve patient outcomes.

The novelty of the manuscript is in describing how gynecologic oncology patients can be managed through an organizational effort that fully integrates clinical practice, innovative facilities, and cutting-edge research within the same infrastructure to meet each patient’s needs. As we seek to move forward with personalized patient management and care, multidisciplinarity leads to an integrated pathway from a systematic approach to a more flexible, patient-driven approach to standards of care.

## Figures and Tables

**Table 1:** List of adopted clinical solutions for specific patients’ needs.

Patients	Clinical Solution	Description
Endometrial cancer	Ultra-minimally invasive approach	Minimally invasive surgery using ultra-thin instruments with the aim of further reducing postoperative pain and improve aesthetic outcomes [72].
All patients	Assessment of nutritional status	Patients are assessed with specially designed tools to guide the potential need for supplements in both the preoperative and postoperative periods [73,74].
All patients	Cinema therapy as supportive care	The psycho-oncologist organizes bi-weekly groups for patients to watch specific movies, and reflect on specific themes related to the illness experience.
All patients	Psycho-oncologists	Patients receive specific questionnaires to assess their Quality of Life (QoL), while having the possibility to talk with the psycho-oncologist about their illness experience (e.g., EORTC QLQ-OV28, EORTC QLQ-OV30, EORTC QLQ-CR29, EQ-5D-5L) [75].
Obese patients (BMI > 30)	Access to robotic platform	Patients are given the opportunity to be operated on with the robotic approach, which has been demonstrated as beneficial by different studies in the literature [76].
Ovarian and endometrial cancer patients	Molecular profiling of tumors as a standard for risk definition	Possibility to characterize endometrial and ovarian tumors from a molecular perspective to tailor adjuvant treatment. For other cancers, specific research protocols are available.
Patients with personal or familiar history of multiple cancers	Genetic profiling and counselling	Patients with personal/familiar history of multiple cancers or according to the patient’s age are tested for genetic profiling and given individualized counselling.
Young patients (<40 years)	Fertility-sparing treatment in early-stage disease	Young patients with early-stage disease can be treated with uterine conservation leaving the possibility of subsequent pregnancy [77,78]. A multidisciplinary team (gynecologists, psychologists, oncologists, senologists, ematologists, radiation oncologists, and pediatricians) provides specific patient-specific counselling to identify viable candidates.
Elderly patient (>75 years)	Specialized geriatrician	Patients receive a specific questionnaire (G8) to assess their frailty score [79].
Patients from other regions/unable to travel for long distances	Telemedical consultations	Because many patients had difficulty travelling to the hospital during the COVID-19 pandemic, telemedical consultations with clinicians were arranged to provide continuity of care and maintain contacts between the unit and patients in other regions, to reduce their travel expenses.

**Table 2:** Performance of the Gynecologic Oncology Unit at FPG in 2020.

Total Numbers	2020
Beds	81
Patients treated	4.296
Surgical procedures	4.726
Mininvasive surgical robotic treatments	199
Chemotherapies	13.612
Requested radiotherapies	926
Tumor boards (once a week, subdivided for main pathology sites)	over 140
Active clinical trials	over 40

## Data Availability

Not applicable.
